# Ambient Documentation Technology in Clinician Experience of Documentation Burden and Burnout

**DOI:** 10.1001/jamanetworkopen.2025.28056

**Published:** 2025-08-21

**Authors:** Jacqueline G. You, Reema H. Dbouk, Adam Landman, David Y. Ting, Sayon Dutta, Julie C. Wang, Amanda J. Centi, Molly Macfarlane, Eran Bechor, Jonathan Letourneau, Gabrielle Choo-Kang, Esther H. Kim, Cordula Magee, Brian J. Lang, Laura Angelo, Jackson Olin, Michelle Frits, Christine Iannaccone, Angela Rui, Ivana Salmikova, Christopher Holland, Bryan Blanchette, Rachel Silverman, David W. Bates, Lisa Rotenstein, Rebecca G. Mishuris

**Affiliations:** 1 Digital, Mass General Brigham, Somerville, Massachusetts; 2 Division of General Internal Medicine and Primary Care, Department of Medicine, Brigham and Women’s Hospital, Boston, Massachusetts; 3 Department of Medicine, Emory University School of Medicine, Atlanta, Georgia; 4 Harvard Medical School, Boston, Massachusetts; 5 Department of Emergency Medicine, Brigham and Women’s Hospital, Boston, Massachusetts; 6 Division of General Internal Medicine, Department of Medicine, Massachusetts General Hospital, Boston; 7 Department of Emergency Medicine, Massachusetts General Hospital, Boston; 8 Northeastern University, Boston, Massachusetts; 9 Emory Healthcare, Atlanta, Georgia; 10 Divisions of General Internal Medicine and Clinical Informatics, Department of Medicine, University of California, San Francisco

## Abstract

**Importance:**

Documentation burden is associated with clinician burnout. To address documentation burden, Mass General Brigham (MGB) in Somerville, Massachusetts, and Emory Healthcare in Atlanta, Georgia, have piloted ambient documentation technology (ADT), which develops artificial intelligence–drafted clinical notes from clinician-patient conversations.

**Objective:**

To examine the prevalence of ADT use and its association with clinicians’ experience of documentation burden and burnout before and after use.

**Design, Setting, and Participants:**

This survey study included clinicians who used ADT for at least 42 days and responded to both the pre- and postsurveys (onboarded February 27 to July 31, 2024, at MGB and July 19, 2023, to July 31, 2024, at Emory).

**Exposure:**

Ambient documentation technology.

**Main Outcomes and Measures:**

The main outcomes were the proportion of self-reported ADT usage, change in proportion of clinicians meeting the criteria for burnout or a positive outcome of their documentation practice associated with their well-being, and free-text survey comments.

**Results:**

Across MGB and Emory, 1430 clinicians, including 873 at MGB (573 clinicians [65.6%] practiced for >10 years; 478 women [54.8%]) and 557 at Emory (286 clinicians [51.3%] practiced for >10 years; 309 women [55.5%]) were enrolled in ADT pilot studies. The majority of clinicians were physicians (688 [78.8%] at MGB and 418 [75.0%] at Emory). Approximately equal proportions of clinicians practiced in primary care (192 [22.0%] at MGB and 156 [28.0%] at Emory). The response rates to relevant components of the MGB survey was 30.4% (265 of 873 respondents) for the 42-day midsurvey and 22.0% (192 of 873 respondents) for the 84-day postsurvey; for the Emory survey, the postsurvey response rate was 11.1% (62 of 557 respondents). A total of 128 of 264 MGB survey respondents (48.5%) had self-reported using ADT for at least 50% of their patient visits, while 27 of 62 Emory respondents (43.5%) self-reported usage for most or all of their visits. The proportion of MGB clinicians reporting burnout (Professional Fulfillment Index overall burnout score >1.33) decreased from 50.6% to 29.4% (χ^2^ = 42.4; *P* < .001) at 42 days and decreased from 52.6% to 30.7% (χ^2^ = 32.7; *P* < .001) at 84 days. The proportion of Emory clinicians reporting a positive impact of their documentation practice on well-being (defined as a score of 3-4 for positive or very positive) improved from 1.6% to 32.3% (χ^2^ = 19.0; *P* < .001). Qualitative analysis of free-text survey comments revealed that clinicians found improved joy in practice with ADT use and saw opportunities for improved ADT functionality.

**Conclusions and Relevance:**

Across these survey studies, ADT use was associated with reductions in burnout and increases in perceived well-being. These findings suggest that ADT may enhance clinicians’ documentation-related experience and reduce burnout.

## Key Points

**Question:**

Is the use of ambient documentation technology (ADT) associated with changes in clinician experience of documentation burden and burnout?

**Findings:**

In this survey study of 1430 clinicians from 2 academic medical center systems, ADT was associated with reductions in burnout and improved well-being scores compared with baseline.

**Meaning:**

These findings suggest that ADT may hold promise for enhancing clinicians’ perceived documentation-related well-being and reducing burnout.

## Teaser

This survey study examines clinicians’ experience of documentation burden and burnout before and after using ambient documentation technology to draft clinical notes.

## Introduction

Insufficient time for documentation in the electronic health record (EHR) by clinicians has been associated with a higher odds of burnout,^1^ which in turn is associated with worse patient care quality and safety.^2,3^ Existing tools to address documentation burden include in-person and virtual scribes, documentation workflow changes, documentation education, and natural language processing and other technologies.^4^ Speech recognition, or dictation software, is widely used but has not clearly impacted documentation time or shown downstream effects on clinician outcomes or economic benefits.^5^ While virtual scribes are associated with reductions in clinician time in the EHR,^6^ cost and scribe turnover limit broader implementation. Policy reform, such as changes to Centers for Medicare & Medicaid Services coding requirements, has minimally impacted documentation time.^7,8,9^

Ambient documentation technology (ADT), including ambient documentation or artificial intelligence (AI) scribes, listens to a clinician-patient conversation and drafts a structured note using generative AI (eFigure 1 in Supplement 1). Prior work has shown that ADT may improve overall time in writing notes per appointment,^10^ productivity, clinician time spent per patient, and documentation deficiency rates.^11,12,13^ Single-site, single-vendor studies have shown varying outcomes of ADT associated with clinician experience.^11,14,15^ Some studies have shown improvements in burnout but not professional fulfillment.^12^ Others have shown improvements in clinician disengagement but not burnout.^11^ Overall, there is a need for a broader understanding of the outcomes of ADT associated with multiple aspects of clinician experience across clinician types and specialties. Additionally, aside from results from a single-site pilot study characterizing improvements in administrative burden with ADT,^16^ there is a relative dearth of qualitative data regarding user experience with ADT across specialties.

We report the results of 2 institutions’ survey studies regarding changes in clinician (ie, physician and advanced practice practitioner [APP]) experience with using ADT. We aimed to answer 3 questions: (1) What is the self-reported prevalence of ADT use, (2) how is use of ADT associated with changes in measures of clinician experience, and (3) what are the areas of opportunity for improvement in ADT identified by pilot users?

## Methods

### Study Settings and Interventions

Two survey studies took place at 2 academic medical centers, Mass General Brigham (MGB) in Somerville, Massachusetts, and Emory Healthcare in Atlanta, Georgia. Both systems use Epic (Epic Systems) as their EHR vendor. Both the MGB and Emory University Institutional Review Boards determined that this project did not constitute human participant research. As these were quality improvement studies, informed consent was waived. The study followed the American Association for Public Opinion Research (AAPOR) reporting guideline.

Mass General Brigham is a quaternary academic medical center health care system with 12 hospitals and 7500 physicians. It conducted a nonrandomized, unmasked pilot study with 2 fully AI–based ADT vendors (1 with ambulatory EHR integration and 1 nonintegrated initially), with onboarding starting February 27, 2024, and onward and an intended assessment period of 42 and 84 days. Pilot participants included attending physicians and APPs at MGB and MGB affiliates across ambulatory, emergency department, and inpatient care settings and most specialties except radiology and pathology. Vendor assignment was based on care setting, device type, qualified bilingual speaker status, and specialty leadership input. If users switched platforms due to pragmatic crossover, we categorized them under the assigned vendor platform at the 42-day time point.

Emory Healthcare, which has 11 hospitals and 3400 physicians, initiated a nonrandomized, unmasked, rolling implementation pilot study of 1 fully AI–based EHR-integrated ADT vendor on July 19, 2023. The pilot had no prespecified duration. Project leadership solicited initial participants from multiple specialties. Pilot participants included attending physicians, residents and fellows, and APPs affiliated with Emory’s academic medical centers and academic faculty practices and community practices. Once these participants determined that the clinical output was appropriate and the tool was helpful for their specialty-specific workflow and documentation requirements, all clinicians within that specialty were invited to participate.

### Survey Methodology and Study Sample

Mass General Brigham administered the following surveys to all clinicians piloting ADT: a baseline 23-question intake survey, a 32-question presurvey, a 39-question midpoint survey at the 42-day mark, and a 71-question postsurvey at the 84-day mark using REDCap (Research Electronic Data Capture).^17,18^ The current analysis focused on participants onboarded to the ADT pilot from February 27, 2024, to July 31, 2024. All Emory clinicians piloting ADT were administered a 5-question presurvey and a 9-question postsurvey at the 60-day mark using Microsoft Forms in Microsoft 365 Enterprise (Microsoft Corporation). The current analysis focused on participants onboarded to the ADT pilot from July 19, 2023, to July 31, 2024 (both institutions’ survey instruments are provided in the eMethods in Supplement 1). Participants received email reminders to complete the surveys. Survey participation was voluntary and not compensated. To facilitate comparison of survey results across sites, we compared presurvey results at each institution with those from the 42-day midsurvey and 84-day postsurvey at MGB and the 60-day postsurvey at Emory.

### Data Elements

#### Demographic Information

Demographic data (years practiced, specialty, medical group, gender [man, woman, other (nonbinary, something else, did not understand the question, prefer not to answer), or unknown] or sex [Emory], and clinician role) were obtained from the intake form at MGB and retroactively obtained by the operational team at Emory. Data on race and ethnicity were not collected. Both institutions’ clinical workload data were obtained from Signal^19^ data, an EHR vendor–based practitioner efficiency database. For the MGB cohort, clinicians were asked to self-identify a primary specialty from a list of 75 subspecialties on the intake survey. For the Emory cohort, clinician specialty was retroactively obtained by the operational team (B.B.). These specialties were subsequently grouped into 1 of 5 categories for reporting in this article: primary care, urgent care or emergency medicine, hospitalist, surgery, and other subspecialties (definitions of specialties included in the 5 categories are provided in eTable 1 in Supplement 1).

#### Quantitative Assessment of Clinician Experience

Mass General Brigham measured burnout using the Professional Fulfillment Index (PFI), a validated survey of clinician well-being used nationally, with scoring done on a 5-point Likert scale (0-4).^20^ Burned out was defined as an overall burnout score of greater than 1.33.^20,21,22,23^ Professionally fulfilled was defined as a PFI score of greater than or equal to 3.^20^ Intent to leave was defined as a score greater than or equal to 2, consistent with prior literature.^20,21^ We also assessed EHR experience using 6 survey questions, 4 of which were provided by the Physician Wellness Academic Consortium^24^ and 2 that were internally developed, which were also assessed on a scale of 0 to 4. Likelihood to recommend ADT to another clinician was assessed on the 84-day postsurvey (scale of 1-10). The MGB survey results were included for analyses if, for a given participant, no more than 1 burnout subscale was incomplete in the burnout subsection of the surveys.

Emory assessed well-being with a single question on the overall perceived impact of one’s documentation process on well-being, with answer options provided on a 5-point Likert scale (0-4). A positive well-being score was defined by the Emory team as greater than or equal to 3 (positive or very positive). Well-being survey results were included for analyses if both the presurvey and postsurvey were completed by a given participant. Positive scores for documentation process needs being met and for documentation ease were defined as a score greater than or equal to 3 (agree or strongly agree). Likelihood to recommend to a colleague or coworker was also asked (0-10 scale, transformed to 1-10). Finally, the usability of the ADT tool was assessed using the 2 questions from the Usability Metric for User Experience Lite survey.^25^

#### ADT Usage

Given a lack of standardization in quantifying ADT usage compared with EHR visit volume across institutions, specialties, and clinical schedules and technical limitations in collecting these data, we queried participants on self-reported ADT usage. Participants at MGB were asked to describe usage by proportion of use (no use, ≤24% of visits, 25%-49% of visits, 50%-74% of visits, or ≥75% of visits). Emory users were similarly asked to report usage as either none, few, some, most, or all notes. Self-reported estimated usage was derived from Emory postsurvey data at the 60-day mark and MGB midsurvey data at the 42-day mark.

#### Qualitative Data

For survey participants at both institutions, free-text survey comments were extracted from all surveys for qualitative analysis. At MGB, clinicians were optionally asked to provide other positive or negative feedback in the midsurvey. At Emory, if clinicians responded in the postsurvey that they used ADT for only some, few, or none of their notes, they were asked to explain why.

### Qualitative Analysis

Two researchers from the Brigham and Woman’s Hospital research team (A.R., G.C.K.) and 1 from Emory (I.S.) conducted a qualitative analysis of the survey response comments from MGB’s midsurvey and Emory’s postsurvey questions using a grounded theory inductive approach.^26^ The reviewers were masked to technology vendors. Where needed, 1 author (J.G.Y.) clarified clinical context (eg, medical abbreviations and terms) and workflow and interface context on specific survey responses. Anonymous survey responses along with clinician role and specialty were imported into an Excel spreadsheet in Microsoft 365 for Enterprise (Microsoft Corporation) for data analysis and management. Three authors (A.R., I.S., and G.C.K.) read all survey comments and completed a pilot set of coded comments, independently coding 25 comments, 15 from MGB and 10 from Emory. A codebook of 12 codes (eTable 2 in Supplement 1) was developed by the 3 researchers; 2 (A.R. and I.S.) independently coded remaining comments with adjudication by the third (G.C.K.) as needed. The reviewers summarized the findings for each code by analyzing the comments associated with a code, determining key concepts, and identifying relevant quotes. The reviewers identified relationships across codes to create themes representing clinicians’ experiences.

### Statistical Analysis

Although both surveys sought to gain insight into clinician experience, the survey studies were analyzed separately given that the associated implementations differed (including independent vendor selection and internal assessment processes), and different elements of clinician experience (burnout and well-being) were assessed. Frequencies and percentages were calculated for all categorical variables while medians and IQRs were calculated for all continuous variables. The χ^2^ and Wilcoxon rank sum tests were performed to compare the distributions of variables between survey respondents and nonrespondents, with Fisher exact tests substituting for χ^2^ tests when any cells were less than 6. To compare pre- and postsurvey responses, McNemar and Wilcoxon signed rank tests were performed on dichotomous and continuous variables, respectively. McNemar exact tests were used when stratifying the analyses by ADT usage to account for the possibility that a discordant cell could equal 0. *P* < .05 was considered statistically significant. All analyses were performed using SAS, version 9.4 (SAS Institute Inc). Data were analyzed from October 23 to December 22, 2024.

## Results

Across MGB and Emory, 1430 clinicians, including 873 at MGB (572 clinicians [65.6%] practiced for >10 years; 367 self-identified as men [42.0%], 478 as women [54.8%], and 28 as other or unknown gender [3.2%]) and 557 at Emory (286 clinicians [51.3%] practiced for >10 years; 309 female [55.5%] and 248 male [44.5%]) were enrolled in ADT pilots (Table 1). At MGB, 192 users (22.0%) were primary care clinicians, 209 (23.9%) were surgeons, 84 (9.6%) were urgent care or emergency medicine clinicians, 28 (3.2%) were hospitalists, and 360 (41.2%) were from other specialties. A total of 688 users (78.8%) were physicians and 185 APPs (21.2%). At Emory, 156 users (28.0%) were primary care clinicians, 116 (20.8%) were surgeons, 100 (18.0%) were urgent care or emergency medicine clinicians, 15 (2.7%) were hospitalists, and 170 (30.5%) were from other specialties. Among users at Emory, 418 (75.0%) were physicians and 139 (25.0%) were APPs.

**Table 1.** Participant and Survey Respondent Characteristics

Characteristic	Overall (n = 873), No. (%)	Midsurvey respondents, No. (%)^a^	*P* value^b^	Postsurvey respondents, No. (%)^c^	*P* value^b^
**Mass General Brigham**					
No. of participants or respondents	873	265 (30.4)	NA	192 (22.0)	NA
Years practiced					
1-3	62 (7.1)	21 (7.9)	.62	17 (8.9)	.28
4-6	87 (10.0)	25 (9.4)		15 (7.8)	
7-10	146 (16.7)	41 (15.5)		28 (14.6)	
11-15	191 (21.9)	68 (25.7)		43 (22.4)	
16-20	114 (13.1)	33 (12.5)		30 (15.6)	
>20	267 (30.6)	76 (28.7)		59 (30.7)	
Unknown	6 (0.7)	1 (0.4)		0	
Specialty group					
Primary care	192 (22.0)	69 (26.0)	.003	52 (27.1)	.20
Urgent care or emergency medicine	84 (9.6)	17 (6.4)		14 (7.3)	
Hospitalist	28 (3.2)	5 (1.9)		3 (1.6)	
Surgery	209 (23.9)	50 (18.9)		34 (17.7)	
Other subspecialty^d^	360 (41.2)	124 (46.8)		85 (44.3)	
Medical group					
Academic medical center	449 (51.4)	131 (49.4)	.49	99 (51.6)	.84
Specialty hospital	84 (9.6)	30 (11.3)		22 (11.5)	
Community or other^e^	340 (38.9)	104 (39.2)		71 (37.0)	
Gender					
Man	367 (42.0)	106 (40.0)	.54	85 (44.3)	.84
Woman	478 (54.8)	152 (57.4)		102 (53.1)	
Other or unknown^f^	28 (3.2)	7 (2.6)		5 (2.6)	
Clinician roles					
Physician	688 (78.8)	209 (78.9)	.98	154 (80.2)	.61
Advanced practice practitioner	185 (21.2)	56 (21.1)		38 (19.8)	
Clinical workload					
Ambulatory appointments per day (Signal^19^), median (IQR) (n = 695; n = 232)^g^	9.4 (6.6-13.0)	9.1 (7.0-12.8)	.96	8.9 (6.9-12.3)	.99
No. of patients per day (Signal^19^), median (IQR) (n = 145; n = 26)^g^	8.1 (3.9-12.6)	8.6 (4.0-15.6)	.46	10.0 (5.5-15.8)	.24
**Emory Healthcare**					
No. of participants and respondents	557	NA	NA	62 (11.1)	NA
Years practiced		NA	NA		
1-3	49 (8.8)	NA	NA	1 (1.6)	.15
4-6	93 (16.7)	NA	NA	10 (16.1)	
7-10	129 (23.2)	NA	NA	16 (25.8)	
11-15	120 (21.5)	NA	NA	12 (19.4)	
16-20	70 (12.6)	NA	NA	7 (11.3)	
>20	96 (17.2)	NA	NA	16 (25.8)	
Specialty group					
Primary care	156 (28.0)	NA	NA	13 (21.0)	.001
Urgent care or emergency medicine	100 (18.0)	NA	NA	6 (9.7)	
Hospitalist	15 (2.7)	NA	NA	3 (4.8)	
Surgery	116 (20.8)	NA	NA	25 (40.3)	
Other subspecialty^d^	170 (30.5)	NA	NA	15 (24.2)	
Sex					
Female	309 (55.5)	NA	NA	31 (50.0)	.42
Male	248 (44.5)	NA	NA	31 (50.0)	
Medical group					
Academic medical center	260 (46.7)	NA	NA	30 (48.4)	.96
Specialty hospital	12 (2.2)	NA	NA	1 (1.6)	
Community or other^e^	285 (51.2)	NA	NA	31 (50.0)	
Clinician roles					
Physician	418 (75.0)	NA	NA	49 (79.0)	.53
Advanced practice practitioner	139 (25.0)	NA	NA	13 (21.0)	
Clinical workload					
Ambulatory appointments per day (Signal^19^), median (IQR) (n = 340; n = 46)^g^	9.4 (6.9-12.6)	NA	NA	9.2 (7.1-12.1)	.76
No. of patients per day (Signal^19^), median (IQR) (n = 107; n = 8)^g^	10.2 (7.9-13.7)	NA	NA	10.8 (5.1-11.6)	.58

Abbreviation: NA, not applicable.

^a^ Mass General Brigham survey at 42 days in the study.

^b^ The χ^2^ test was used to compare distributions of categorical variables of survey respondents with nonrespondents at each time point. Fisher exact test was used when cells were less than 6. Wilcoxon rank sum test was used for continuous variables.

^c^ Mass General Brigham postsurvey at 84 days and Emory postsurvey at 60 days.

^d^ Other subspecialties listed in eTable 1 in Supplement 1.

^e^ Other medical groups included community or other affiliates.

^f^ Other gender categories included nonbinary, something else, do not understand the question, or prefer not to answer.

^g^ Ambulatory appointments were averaged for outpatient clinicians only, and number of patients was averaged for inpatient clinicians only.

A total of 265 of the 873 MGB participants responded to both the presurvey and the 42-day midsurvey (30.4% response rate), and 192 responded to both the presurvey and 84-day postsurvey (22.0% response rate). Emory had 179 presurvey responses and 117 postsurvey responses, with 62 survey responses for which survey matching could be performed (11.1% response rate based on matched responses compared with the overall cohort of 557 clinicians) (Table 1).

A total of 128 of the 264 MGB survey respondents (48.5%) had self-reported usage greater than or equal to 50% of visits, while 27 of the 62 Emory respondents (43.5%) had self-reported usage for most or all of their notes (Table 2). The MGB respondents had a median likelihood-to-recommend score of 8.0 (IQR, 6.0-9.5), and Emory had a median likelihood-to-recommend score of 8.0 (IQR, 6.0-10).

**Table 2.** Survey Responses for Self-Reported Estimated Usage and Likelihood to Recommend Ambient Documentation Technology

Estimated usage	Responses, No. (%)
**Mass General Brigham** ^a^	
Estimation of use of AI-generated drafts (n = 264)	
I have not used AI-generated draft notes	4 (1.5)
≤24% of visits	93 (35.2)
25%-49% of visits	39 (14.8)
50%-74% of visits	45 (17.1)
≥75% of visits	83 (31.4)
Overall likelihood to recommend, median (IQR) (n = 148)^b^^,^^c^	8.0 (6.0-9.5)
**Emory Healthcare** ^d^	
Estimation of use of AI-generated drafts (n = 62)	
None of my notes	7 (11.3)
Few of my notes	12 (19.4)
Some of my notes	16 (25.8)
Most of my notes	16 (25.8)
All of my notes	11 (17.7)
Overall likelihood to recommend, median (IQR)^b^	8.0 (6.0-10.0)

Abbreviation: AI, artificial intelligence.

^a^ Midsurvey responses at 42 days.

^b^ Likelihood-to-recommend scale of 1 to 10, with higher numbers indicating more likely to recommend.

^c^ Postsurvey responses at 84 days.

^d^ Postsurvey responses at 60 days.

At MGB, the number of respondents meeting the criteria for professional fulfillment increased at both the 42-day mark and 84-day mark but was not statistically significant, increasing from 90 (34.0%) to 99 (37.4%) (χ^2^ = 1.2; *P* = .26) at 42 days and from 57 (29.7%) to 67 (34.9%) (χ^2^ = 2.5; *P* = .11) at 84 days. The number of MGB respondents meeting the criteria for burnout decreased significantly from 134 (50.6%) at baseline to 78 (29.4%) (χ^2^ = 42.4; *P* < .001) at 42 days and from 101 (52.6%) at baseline to 59 (30.7%) (χ^2^ = 32.7; *P* < .001) at 84 days. At MGB, changes to the number of clinicians with intentions to leave was not statistically significant at 42 days (from 66 [24.9%] to 72 [27.2%]; χ^2^ = 0.9; *P* = .35) and 84 days (from 54 [28.3%] to 51 [26.6%]; χ^2^ = 0.3; *P* = .61). Meanwhile, the number of Emory clinicians reporting their documentation process as having a positive impact on well-being increased significantly from 1 (1.6%) to 20 (32.3%) (χ^2^ = 19.0; *P* < .001) after 60 days (Table 3). Analyses comparing burnout and well-being, as well as EHR experience scores, as ordinal variables are provided in eTable 3 in Supplement 1. Changes in burnout and well-being frequencies by ADT usage level and by specialty are shown in eTables 4 and 5, respectively, in Supplement 1.

**Table 3.** Survey Responses for Burnout and Well-Being

Scale^a^	Responses, No. (%)			*P* value^b^
	Presurvey	Midsurvey	Postsurvey	
**Mass General Brigham (n = 265)**				
Professionally fulfilled	90 (34.0)	99 (37.4)	NA	.26
Burned out	134 (50.6)	78 (29.4)	NA	<.001
Intention to leave	66 (24.9)	72 (27.2)	NA	.35
Overall (n = 192)				
Professionally fulfilled	57 (29.7)	NA	67 (34.9)	.11
Burned out	101 (52.6)	NA	59 (30.7)	<.001
Intention to leave	54 (28.3)	NA	51 (26.6)	.61
**Emory Healthcare (n = 62)**				
Positive impact on well-being	1 (1.6)	NA	20 (32.3)	<.001

Abbreviation: NA, not applicable.

^a^ Professional Fulfillment Index burnout and well-being dichotomized cutoff scores for Mass General Brigham: professionally fulfilled, 3 or higher; burned out, 1.33 or higher; intention to leave, 2 or higher. Emory Healthcare cutoff scores: 3 or higher for each scale.

^b^ From McNemar test for comparing dichotomous variables.

Our analysis of qualitative free-text comments included 139 clinician responses from MGB and 20 from Emory, with the top 5 categories of feedback encompassing AI note style, usefulness, satisfaction, efficiency and time, and workload. As shown in Table 4, some users indicated that ADT improved their clinic experience and subjective patient experience, with 1 responding that more “contact with patients and families…definitely makes clinic easier.” Others indicated that the technology “improves [users’] joy in practice” and addressed documentation needs, with “the assessment and plan [being] more complete than what [the user] would have documented.” For some respondents, this technology has the potential to “fundamentally [change] the experience of being a physician.” However, some noted that the ADT had less utility for certain visit and content types, such as pediatric physical examination visits and psychiatry visits. There was mixed commentary around efficiency and time, with some users indicating additional time to work on other tasks, while others found “it added 1 to 2 hours a day to my note writing.” Other categories of feedback and corresponding frequencies are included in eTable 6 in Supplement 1.

**Table 4.** Free-Text Comment Analysis

Theme	Respondents, No. (%)			Representative user comments
	Overall (n = 159)	MGB (n = 139)	Emory Healthcare (n = 20)	
AI note style	83 (52.2)	71 (51.1)	12 (60.0)	“The AI-generated text is also superfluous, leading to bulky notes. This increases the amount of time other care team members have to read notes. Errors tend to be minor but are frequent. Does not capture visits in Spanish well. I am bilingual, so not sure if it is related to lack of use of interpreter.” (MGB, primary care internal medicine) “Most of my visits are physical exams as a pediatrician and do not work well for this visit type. Organization not good. Should have sections for diet, elimination, development, sleep, home environment, activities, school, dental, vision, hearing, and growth and anticipatory guidance and should have problem-based section for addressing chronic problems and health care maintenance. I basically use to make sure I don’t forget anything in my note but then use my usual template.” (MGB, primary care pediatrics)
Usefulness	63 (39.6)	59 (42.4)	4 (20.0)	“Exceptionally helpful. Definitely improves my contact with patients and families, and definitely makes clinic easier. I still need to go through and edit the note, particularly the exam, but on the other hand, the HPI as well as the assessment and plan are often more complete than what I would have documented.” (MGB, neurology) “It doesn’t capture goals-of-care discussions really at all or psychosocial or spiritual health discussions, and the symptom management notes are shorter anyways, so hasn’t helped me all that much. It is most helpful for new patient visits, I think. Allows me to focus on the conversation a bit more than I might otherwise be able to.” (Emory Healthcare, hospice and palliative care)
Satisfaction	56 (35.2)	51 (36.7)	5 (25.0)	“It definitely improves my joy in practice because I get to interact with patients and look them in the eye without worrying I will forget what they are saying later. As the tools grow, I think they will fundamentally change the experience of being a physician.” (MGB, infectious diseases) “I’m not ready to hand my documentation over to AI yet, but I do believe this has the potential to significantly reduce note burden. Integration is clearly the biggest drawback currently. But there is also room for improvement on organization of problem-based charting in the note proper.” (MGB, primary care internal medicine)
Efficiency and time	42 (26.4)	38 (27.3)	4 (20.0)	“Because of using [vendor], I get about an hour back every day that I can use to call patients back before 7 or 8 pm, respond to gateway, etc.” (MGB, primary care internal medicine) “I tried my best with it but found it added 1 to 2 hours a day to my note writing, [which] meant I left clinic with fewer notes completed, had no actionable information in them.” (MGB, pulmonology)
Workload	38 (23.9)	35 (25.2)	3 (15.0)	“It is a nuisance to copy and paste and do all the editing, but that is somewhat mindless work, whereas actually creating the text from scratch hours later requires more thinking. In the afternoon, I don’t focus as well and when busy charts will stack up. I no longer have the bandwidth to do the notes. Having them already half done is great.” (MGB, general surgery) “For routine follow-ups, the way I use [EHR] works very well, and little time is spent with documentation largely because of flexible copy-forward options and autotext shortcuts. For new patients and outpatient consults, my interviews are very detailed, I collect a lot of information, and there is a rich discussion with the patient about treatment planning and options. In my experience, [vendor] does a very poor job of summarizing this, often boiling down a 90- to 120-minute visit to just a few sentences. The bigger problem is that [vendor] is just not designed with psychiatry in mind, so even the summaries it provides for routine visits are hit-or-miss in terms of accurately summarizing what I feel is important about the visit.” (Emory, psychiatry)

Abbreviations: AI, artificial intelligence; EHR, electronic health record; HPI, history of present illness; MGB, Mass General Brigham.

## Discussion

In this analysis of survey studies from 2 geographically distinct academic medical centers, we found ADT to be associated with improvements in clinician experience. Clinician experience is multifaceted, and we focused on 2 key aspects of clinician experience: burnout and well-being associated with documentation. Specifically, at MGB, ADT use was associated with a 21.2% absolute reduction in burnout prevalence, while at Emory, ADT use was associated with a 30.7% absolute increase in documentation-related well-being prevalence. While promising, given limited survey response rates, these findings may represent the experience of more enthusiastic users and a best-case scenario for the outcomes associated with ADT.

US clinicians face persistent threats to their experience, with nearly one-half of physicians reporting burnout in 2023^27^ and similar concerns for burnout among physician assistants and nurse practitioners.^28,29^ Organizations have struggled to implement interventions that sustainably enhance the clinician experience.^30^ Our results, representing clinician experiences from multiple sites and specialties and testing multiple vendors, suggest that ADT may be an effective approach to enhancing clinician experience.

Our study adds to the evidence from recent studies on ADT by describing multiple facets of clinician experience, both qualitatively and quantitatively assessing changes in experience, including nonambulatory clinicians at both institutions, and studying a significantly larger sample of clinicians at MGB than has been previously studied. For example, in a previous qualitative study, Shah et al^15^ at Stanford found significant improvements in task load, burnout, and usability in a cohort of 38 physicians (25 primary care physicians and 13 ambulatory specialty physicians). Meanwhile, Misurac et al^14^ at The University of Iowa observed significant reductions in median PFI burnout scores but not professional fulfillment in a cohort of 35 physicians and APPs (including primary care and other subspecialties). A study conducted by Owens et al^11^ at University of Michigan Health West in 83 primary care physicians and APPs did not find overall improvements in burnout. A study looking at an earlier, smaller cohort of the clinicians at Emory also found that ADT was associated with improvements in documentation-related well-being.^31^ Our findings corroborate positive outcomes associated with this technology in a large cohort across multiple institutions while considering multiple aspects of clinician experience.

Less than half of clinicians at MGB reported using ADT at least 50% of the time, and a similar proportion at Emory reported using the technology most or all of the time. This self-reported usage underscores that there are visit types for which ADT may not yet be optimized, such as well-child visits and psychiatry encounters. To improve users’ ADT experience, implementation teams should identify visit types optimal for ADT, and vendors could target other visit types for improvement. Interestingly, despite 35.2% of MGB and 19.4% of Emory survey respondents reporting that they used ADT for few of their visits, use of the technology was associated with reductions in burnout and improvements in perceived documentation-related well-being. Pursuant to prior research showing an association between clinician burnout and cognitive load, this positive perceived outcome may be associated with relief of cognitive burden during encounters in which ADT was used, even if the technology was not optimally useful for all encounters.^32^

Our survey findings and qualitative analysis point to both strengths and areas for improvement for ADT. We saw high end-user satisfaction, with observed median likelihood-to-recommend scores of 8 (out of 10) at both institutions (Table 2). The high ADT satisfaction scores aligned with qualitative comments around ADT’s usefulness and users’ satisfaction with the technology. Notably, the satisfaction scores contrasted with previous research showing poor usability scores for EHRs.^33^ However, some survey comments highlighted workflow and technology limitations, including gaps in specialty-specific content. These themes may guide future enhancements to ADT used in initial pilot studies.

### Limitations

This study has several limitations. Foremost, it was a noncontrolled study of 2 academic medical center systems with a limited number of vendors. The voluntary nature of both institutions’ pilots may have influenced who chose to participate. Academic clinicians in our study may have different clinical volume, documentation patterns, and clinician experience compared with nonacademic clinicians. Intrainstitutional differences in survey respondents compared with overall cohorts were potentially due to differences in user satisfaction with ADT across specialties. We only included respondents with complete pre- and postsurvey results; potentially, clinicians experiencing burnout might have been less likely to respond at all time points. Survey fatigue with longer, more frequent surveys may have limited the survey response rate at MGB, while survey response matching limited the survey response rate at Emory. Given the response rate, it is possible that our findings represent the experience of users with strong opinions and may not represent the experience of all clinicians exposed to ADT. However, the response rate is consistent with the wide range of burnout-related survey responses in the literature, ranging from 17% to 54%.^21,23,34^ We observed a statistically significant difference in subspecialty composition of survey respondents compared with the overall intervention cohorts, with more primary care respondents at MGB and more surgical respondents at Emory. As different specialties have different burnout rates,^35^ this difference may have influenced the responses, although the directionality was unclear. These outcomes represent implementation of ADT with specific AI models and product features at a specific point in time; thus, results may change as this technology evolves. The association of ADT with long-term clinician experience is currently unclear. Finally, measures of ADT use need standardization. Given challenges with measuring ADT use objectively relative to data quality issues, particularly with ADTs that were not integrated with the EHR, we chose to study self-reported usage, which is subject to recall bias. For future research and development, standard and linked metrics for ADT, potentially developed in partnership with EHR and ADT vendors, may improve outcome evaluation in domains such as usage, burnout, cost of turnover, and productivity and enable consistent comparison across health care organizations and over time.

## Conclusions

Across the survey studies at 2 academic medical centers, which were limited by low response rate and, therefore, possibly representing enthusiastic users, ADT was associated with significant reductions in burnout and improvements in perceived documentation-related well-being. Further work is needed to understand how to maximize well-being benefit across clinicians; the sustainability and scalability of the association found; and the outcomes of ADT interventions associated with patient satisfaction and experience, financial sustainability, and generalizability at scale of ADT.

## Notes

Supplement 1. **eFigure 1.** Ambient Documentation Workflow **eFigure 2.** Survey Instrument Administration **eTable 1.** Specialty Categorization **eTable 2.** Quantitative Analysis Codebook **eTable 3.** Continuous Scales of Survey Responses for Burnout, Well-Being, and EHR Experience/Documentation Process **eTable 4.** Survey Responses for Burnout, Well-Being, and Documentation Process by Ambient Usage **eTable 5.** Survey Responses for Burnout, Well-Being, and Documentation Process by Specialty **eTable 6.** Qualitative Analysis Code Counts for MGB and Emory Clinicians **eMethods.** Survey Instruments

Supplement 2.
Data Sharing Statement
