# Supplementary material for: Ambient Documentation Technology in Clinician Experience of Documentation Burden and Burnout
Source: JAMA Netw Open. 2025 Aug 21;8(8):e2528056. doi: 10.1001/jamanetworkopen.2025.28056 (PMC12371510; doi:10.1001/jamanetworkopen.2025.28056)
Supplement: Supplement 1. — eFigure 1. Ambient Documentation Workflow eFigure 2. Survey Instrument Administration eTable 1. Specialty Categorization eTable 2. Quantitative Analysis Codebook eTable 3. Continuous Scales of Survey Responses for Burnout, Well-Being, and EHR Experience/Documentation Process eTable 4. Survey Responses for Burnout, Well-Being, and Documentation Process by Ambient Usage eTable 5. Survey Responses for Burnout, Well-Being, and Documentation Process by Specialty eTable 6. Qualitative Analysis Code Counts for MGB and Emory Clinicians eMethods. Survey Instruments [file jamanetwopen-e2528056-s001.pdf]

Supplemental Online Content

You JG, Dbouk RH, Landman A, et al. Ambient documentation technology in clinician experience of documentation burden and burnout. *JAMA Netw Open*. 2025;8(8):e2528056. doi:10.1001/jamanetworkopen.2025.28056

eFigure 1. Ambient Documentation Workflow

eFigure 2. Survey Instrument Administration

eTable 1. Specialty Categorization

eTable 2. Quantitative Analysis Codebook

eTable 3. Continuous Scales of Survey Responses for Burnout, Well-Being, and EHR Experience/Documentation Process

eTable 4. Survey Responses for Burnout, Well-Being, and Documentation Process by Ambient Usage

eTable 5. Survey Responses for Burnout, Well-Being, and Documentation Process by Specialty

eTable 6. Qualitative Analysis Code Counts for MGB and Emory Clinicians

eMethods. Survey Instruments

This supplemental material has been provided by the authors to give readers additional information about their work.

© 2025 You JG et al. *JAMA Network Open*.

eFigure 1. Ambient Documentation Workflow

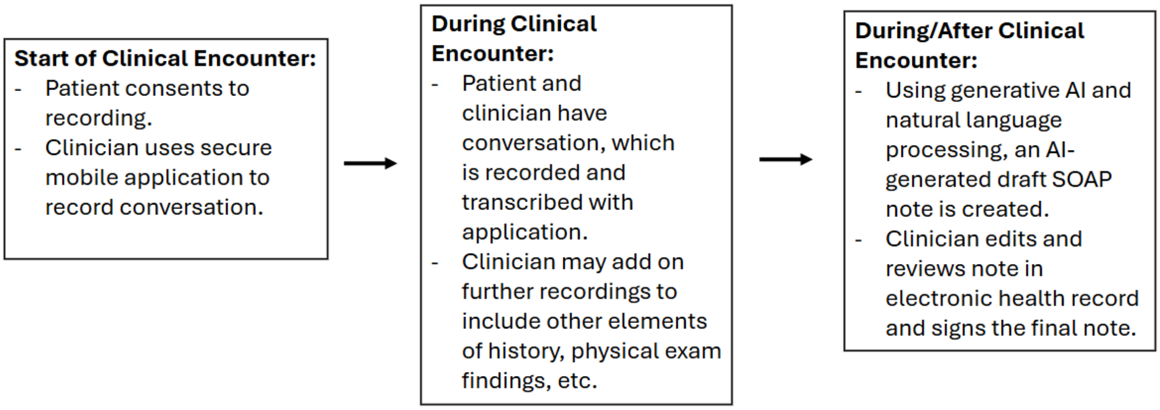

*Vendor variation exists with features such as electronic health record integration, ability to generate an additional patient after visit summary, etc. - the workflow depicted above is generalized.

eFigure 2. Survey Instrument Administration

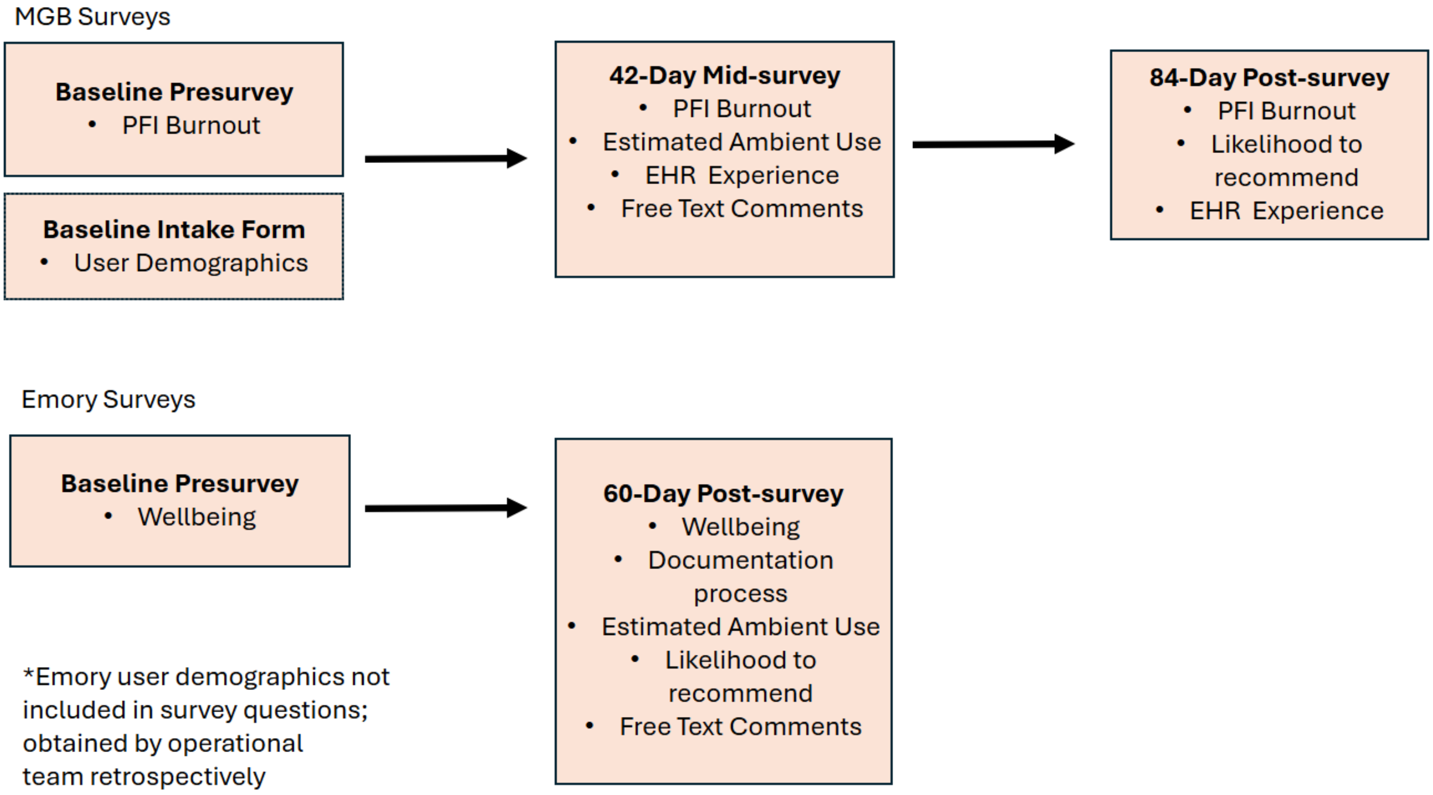

© 2025 You JG et al. *JAMA Network Open*.

eTable 1. Specialty Categorization

Specialty Category	Subspecialties Included
Primary Care	• Primary Care, Internal Medicine<br>• Primary Care, Family Medicine<br>• Primary Care, Pediatrics<br>• Primary Care, Medicine / Pediatrics<br>• Geriatrics<br>• Adolescent Medicine
Urgent Care/Emergency Medicine	• Urgent Care<br>• Emergency Medicine, Adult<br>• Emergency Medicine, Pediatrics
Hospitalist	• Hospital Medicine, Adult<br>• Hospital Medicine, Medicine / Pediatrics<br>• Hospital Medicine, Pediatrics
Surgery	• Anesthesiology<br>• Breast Surgery<br>• Cardiac Surgery<br>• Colorectal Surgery<br>• Endocrine Surgery<br>• General and GI Surgery<br>• Minimally Invasive Surgery<br>• Neurosurgery<br>• Obstetrics & Gynecology (Including Gynecologic Oncology)<br>• Ophthalmology<br>• Oral & Maxillofacial Surgery<br>• Orthopedic Surgery<br>• Otolaryngology (ENT, including Otology, Laryngology, Neurotology)<br>• Pediatric Surgery<br>• Plastic and Reconstructive Surgery<br>• Podiatry<br>• Spine<br>• Surgical Oncology (Cancer Surgery)<br>• Thoracic Surgery<br>• Transplant Surgery<br>• Urology<br>• Vascular Surgery
Other Subspecialties	Mass General Brigham<br>• Addiction Medicine<br>• Allergy and Immunology<br>• Home Hospital<br>• Behavioral Health

© 2025 You JG et al. *JAMA Network Open*.

	• Cardiology (Interventional and Noninterventional)<br>• Dermatology (including Derm Surgery)<br>• Endocrinology, Diabetes, and Metabolism<br>• Gastroenterology (including Hepatology)<br>• Genetics & Genomics<br>• Hematology and Oncology<br>• Infectious Diseases<br>• Nephrology<br>• Neurology<br>• Oral Medicine and Dentistry<br>• Pain Medicine<br>• Palliative & Hospice Care<br>• Pediatric Cardiology<br>• Pediatric Endocrinology<br>• Pediatric Gastroenterology<br>• Pediatric Genetics<br>• Pediatric Neurology<br>• Physical Medicine & Rehabilitation<br>• Psychiatry, Adult/Geriatric<br>• Psychiatry, Child/Adolescent<br>• Pulmonology<br>• Radiation Oncology<br>• Rheumatology<br>• Sleep Medicine<br>• Sports Medicine<br>• Trauma, Burn, Surgical and Critical Care<br>Emory<br>• Adult Congenital Heart Disease<br>• Advanced Heart Failure and Transplant Cardiology<br>• Audiology<br>• Cardiology<br>• Critical Care Medicine<br>• Dermatology<br>• Diagnostic Radiology<br>• Endocrinology<br>• Gastroenterology<br>• Hematology<br>• Hematology and Oncology<br>• Hospice and Palliative Medicine<br>• Infectious Diseases<br>• Interventional Cardiology<br>• Interventional Radiology<br>• Medical Oncology<br>• Nephrology<br>• Neurology<br>• Oncology<br>• Pain Medicine<br>• Palliative Medicine

© 2025 You JG et al. *JAMA Network Open*.

	• Physical Medicine and Rehabilitation<br>• Psychiatry<br>• Pulmonary Disease<br>• Radiation Oncology<br>• Rheumatology<br>• Sleep Medicine<br>• Sports Medicine<br>• Transplant<br>• Transplant Hepatology<br>• Transplant Nephrology

© 2025 You JG et al. *JAMA Network Open*.

eTable 2. Quantitative Analysis Codebook

Codes	Definition	Examples
Efficiency/Time	If the tech saves time, how quickly the provider can complete the appointment using the tech, how many clicks it takes to use specific features	• Time saver<br>• Does not save time: copying and pasting takes more time, editing
Satisfaction	Clinician's reaction to the tech, if they like or dislike it, have they continued using it, if they feel like the technology is at an acceptable standard to be used in clinical care	• Not completely satisfied with tech<br>• Satisfied with quality of tech<br>• Enjoys the tech<br>• Stopped using the program<br>• Continued using the program
Usefulness	Is the technology easy to use, does it have value in specific settings, practical use cases	• Content is not usable<br>• Errors are frequent<br>• Easy to use<br>• More valuable to for shorter visits or specific types of visits<br>• Does not capture other languages well
Accessibility	Anything related to the actual physical use of the technology if there's an ergonomic factor that makes it difficult (voice is hard to recognize by tech, physical limitations like phone battery/internet)	• Mobile/desktop<br>• Logging into the vendor<br>• User has an accent and tech can/cannot accommodate
Impact	If the technology has any positive or negative outcome on clinical care, reducing note burden, how the appointment goes, and time with patient. How does the tech impact the clinician's mental state: does the provider feel better or worse with their tasks after using it?	• Has potential to reduce note burden<br>• Mental demand/burnout: mental state, how much does the clinician have to think w/ using the technology (e.g. this takes too much effort to actually use)<br>• Gives more time with patient. Improves quality of patient interaction
Workload	Whether or not the technology gives the provider more or less work to do, does it require changes in habit, workflow, attention	• Workflow: Difficult to change workflow Additional editing or corrections that have to be made
EHR Integration	How the technology interacts with the EHR, whether the provider needs to upload information into the EHR	• Would be better if could automatically enter problems in EHR<br>• Main reason a lot of users want EHR integration is due to the amount of copy and pasting that needs to be done to upload the note into the patient's chart

© 2025 You JG et al. *JAMA Network Open*.

Patient's Perception	Patient’s opinions and reaction to the technology	• Patients are open to using it
AI Hallucination	Notes are incorrect, out of context, nonsensical	• AI makes something up
AI Note Style	Responses related to the role the AI has in generating the note, quality of information being captured and recorded by AI, different settings and subspecialities having issues with specific medical terms, grouping of problems, issues with pronouns, and recommendation from clinicians on the note style	Specific recommendations or issues having to do with the note:<br>• When the visit is long but the AI summarizes in only a few sentences<br>• Lack of problem-based charting, grouping ideas, how the data is stored, putting separate ideas into the EHR as separate boxes<br>• Psychiatry and dermatology having trouble with getting the AI to recognize specific medical terms<br>• AI being unable to accommodate or learn the providers’ style of notetaking<br>• AI automatically using they/them even when gender is specified<br>Formatting issues:<br>• Would be helpful if it could follow EHR formatting
Errors of Omission	AI fails to include specific data points	• Social history
Training on Technology	Any training requests related to provider interacting with ambient scribing technology	• Need a training course for providers who aren’t used to narrating to scribes

© 2025 You JG et al. *JAMA Network Open*.

eTable 3. Continuous Scales Survey Responses for Burnout, Well-Being, and EHR Experience/Documentation Process

	MGB			Emory			
**Burnout and Wellbeing Continuous Scale (Scale 0-4; median (IQR))**							
**Overall** (n=265)	Pre-survey	Mid-survey, 42 days	P-value*	**Overall** (n=62)	Pre-survey	Post-survey, 60 days	P-value*
Professional Fulfillment	2.5 (2.0, 3.0)	2.7 (2.0, 3.0)	**0.0030**	Impact of documentation on wellbeing	1 (1, 2)	2 (1, 3)	**<0.0001**
Overall Burnout	1.4 (0.9, 1.8)	1.1 (0.6, 1.4)	**<0.0001**				
Intention to Leave	1.0 (0, 1.5)	1.0 (0, 2.0)	0.6405				
EHR experience subscale	2.7 (2.3, 3.0)	2.5 (2.3, 2.8)	0.1025	Documentation process met my requirements as a provider	2 (1, 3)	3 (3, 3)	**0.0010**
				Documentation process was easy for me to complete	2 (1, 3)	3 (2, 3)	**<0.0001**
**Overall** (n=192)	Pre-survey	Post-survey, 84 days	P-value*				
Professional Fulfillment	2.3 (1.8, 3.0)	2.6 (1.8, 3.0)	**0.0388**				
Overall Burnout	1.4 (1.0, 1.9)	1.0 (0.4, 1.4)	**<0.0001**				
Intention to Leave	1.0 (0, 2.0)	1.0 (0, 2.0)	0.4495				
EHR experience subscale	2.7 (2.3, 3.0)	2.6 (2.3, 3.0)	0.2521				

* Wilcoxon Signed-Rank for continuous variables testing null hypothesis that the change from pre- to post-survey is 0

MGB assessed EHR experience using survey questions provided by the Physician Wellness Association Consortium and part of MGB's internal clinician experience survey on a scale of 0-4. Emory assessed usability of ambient documentation using questions from the UMUX (Usability Metric for User Experience) Lite survey.

© 2025 You JG et al. *JAMA Network Open*.

eTable 4. Survey Responses for Burnout, Well-Being, and Documentation Process by Ambient Usage

**MGB**										
**Burnout and Wellbeing Dichotomized Cutoffs by Ambient Usage (n, %)**										
**Self-Reported Usage**	n	Professionally fulfilled			Burned-out			Intention to leave		
**42 Day Comparison**		Pre-survey	Mid-survey, 42 days	P-value*	Pre-survey	Mid-survey, 42 days	P-value*	Pre-survey	Mid-survey, 42 days	P-value*
< 50% of visits	136	45 (33.1%)	50 (36.8%)	0.4421	63 (46.3%)	47 (34.6%)	**0.0070**	35 (25.7%)	43 (31.6%)	0.1516
≥ 50% of visits	128	44 (34.4%)	48 (37.5%)	0.6271	71 (55.5%)	31 (24.2%)	**<.0001**	30 (23.4%)	29 (22.7%)	1.0
**84 Day Comparison**		Pre-survey	Post-survey, 84 days	P-value*	Pre-survey	Post-survey, 84 days	P-value*	Pre-survey	Post-survey, 84 days	P-value*
< 50% of visits	64	19 (29.7%)	23 (35.9%)	0.3437	28 (43.8%)	21 (32.8%)	0.1435	19 (29.7%)	19 (29.7%)	1.0
≥ 50% of visits	83	27 (32.5%)	32 (38.6%)	0.4244	45 (54.2%)	20 (24.1%)	**<.0001**	19 (22.9%)	18 (21.7%)	1.0
**Emory**										
**Wellbeing Dichotomized Cutoffs by Ambient Usage (n, %)**										
**Self-Reported Usage**	n	Positive impact on wellbeing			Documentation process met requirements			Easy documentation process		
		Pre-survey	Post-survey, 60 days	P-value*	Pre-survey	Post-survey, 60 days	P-value*	Pre-survey	Post-survey, 60 days	P-value*
None to some of my notes	35	1 (2.9%)	3 (8.6%)	0.5000	15 (42.9%)	23 (65.7%)	0.0574	8 (22.9%)	14 (40.0%)	0.1460
Most or all of my notes	27	0 (0%)	17 (63.0%)	**0.0020**	14 (51.9%)	24 (88.9%)	**0.0063**	8 (29.6%)	18 (66.7%)	**0.0129**

*From McNemar's exact test for comparing paired dichotomous variables

PFI Cutoffs
Professionally Fulfilled = Professional Fulfillment Index Score ≥ 3
Burned-out = PFI Overall Burnout ≥ 1.33
Intention to leave = PFI Intention to Leave ≥ 2
Emory Cutoffs
Defined as a score of ≥3 for each scale

© 2025 You JG et al. *JAMA Network Open*.

eTable 5. Survey Responses for Burnout, Well-Being, and Documentation Process by Specialty

	MGB				Emory			
**Burnout and Wellbeing Dichotomized Cutoffs by Specialty (n, %)**								
**Specialty**	n	Scale	Pre-survey	Mid-survey, 42 days	n	Scale	Pre-Survey	Post-Survey, 60 days
**Primary Care**	69	Professionally fulfilled	18 (26.1%)	15 (21.7%)	13	Positive impact on wellbeing	0 (0%)	6 (46.2%)
		Burned-out	40 (58.0%)	27 (39.1%)		Documentation process met requirements	8 (61.5%)	11 (84.6%)
		Intention to leave	16 (23.2%)	20 (29.0%)		Easy documentation process	6 (46.2%)	10 (76.9%)
**Urgent Care/ Emergency Medicine**	17	Professionally fulfilled	5 (39.4%)	4 (23.5%)	6	Positive impact on wellbeing	1 (16.7%)	2 (33.3%)
		Burned-out	11 (64.7%)	4 (23.5%)		Documentation process met requirements	3 (50.0%)	3 (50.0%)
		Intention to leave	8 (47.1%)	10 (58.8%)		Easy documentation process	2 (33.3%)	1 (16.7%)
**Hospitalist**	5	Professionally fulfilled	1 (20.0%)	3 (60.0%)	3	Positive impact on wellbeing	0 (0%)	1 (33.3%)
		Burned-out	4 (80.0%)	2 (40.0%)		Documentation process met requirements	3 (100%)	2 (66.7%)
		Intention to leave	2 (40.0%)	2 (40.0%)		Easy documentation process	1 (33.3%)	3 (100%)
**Surgery**	50	Professionally fulfilled	20 (40.0%)	24 (48.0%)	25	Positive impact on wellbeing	0 (0%)	6 (24.0%)
		Burned-out	24 (48.0%)	13 (26.0%)		Documentation process met requirements	9 (36.0%)	19 (76.0%)
		Intention to leave	9 (18.0%)	12 (24.0%)		Easy documentation process	5 (20.0%)	9 (36.0%)
**Other Subspecialty**	124	Professionally fulfilled	46 (37.1%)	53 (42.7%)	15	Positive impact on wellbeing	0 (0%)	5 (33.3%)
		Burned-out	55 (44.4%)	32 (25.8%)		Documentation process met requirements	6 (40.0%)	12 (80.0%)
		Intention to leave	31 (25.0%)	28 (22.6%)		Easy documentation process	2 (13.3%)	9 (60.0%)

**Specialty**	n	Scale	Pre-survey	Post-survey, 84 days
**Primary Care**	52	Professionally fulfilled	9 (17.3%)	13 (25.0%)
		Burned-out	36 (69.2%)	21 (40.4%)
		Intention to leave	15 (28.9%)	12 (23.1%)
**Urgent Care/ Emergency Medicine**	15	Professionally fulfilled	6 (40.0%)	6 (40.0%)
		Burned-out	7 (46.7%)	3 (20.0%)
		Intention to leave	7 (46.7%)	8 (53.3%)
**Hospitalist**	3	Professionally fulfilled	1 (33.3%)	2 (66.7%)
		Burned-out	2 (66.7%)	2 (40.0%)
		Intention to leave	1 (33.3%)	1 (33.3%)

© 2025 You JG et al. *JAMA Network Open*.

**Surgery**	34	Professionally fulfilled	11 (32.4%)	12 (35.3%)
		Burned-out	17 (50.0%)	11 (32.4%)
		Intention to leave	10 (29.4%)	12 (35.3%)
**Other Subspecialty**	88	Professionally fulfilled	30 (34.1%)	34 (38.6%)
		Burned-out	39 (44.3%)	23 (26.1%)
		Intention to leave	21 (23.9%)	18 (20.5%)

No statistical testing done due to small sample sizes when stratified.
PFI Cutoffs
Professionally Fulfilled = Professional Fulfillment Index Score ≥ 3
Burned-out = PFI Overall Burnout ≥ 1.33
Intention to leave = PFI Intention to Leave ≥ 2
Emory Cutoffs
Defined as a score of ≥3 for each scale

© 2025 You JG et al. *JAMA Network Open*.

eTable 6. Qualitative Analysis Code Counts for MGB and Emory Clinicians

Code	Number of survey participants	MGB	Emory
AI Note Style	83	71	12
Usefulness	63	59	4
Satisfaction	56	51	5
Efficiency/Time	42	38	4
Workload	38	35	3
Impact	28	27	1
EHR Integration	23	21	2
Accessibility	10	9	1
AI Hallucination	8	8	0
Patient's Perception	7	7	0
Training on Technology	7	7	0
Errors of Omission	6	3	3

© 2025 You JG et al. *JAMA Network Open*.

eMethods. Survey Instruments

A. MGB Intake Form Text

AI Generated Draft Notes Sign-Up

Thank you for your interest in our pilot. The information collected in this survey will help us set you up for the pilot with our vendors and ensure a range of clinical backgrounds is represented in our pilot. The pilot will last X months and, on average require about X minutes per month for feedback-related activities. By filling out this form, you agree to participate in our pilot, which includes:

Introduction to technology and setup (30min Teams meeting)

- Pre, mid-point and post-survey (via RedCap)
- Optional in-depth, qualitative interview about documentation habits
- Individual outreach as needed to address concerns
- Group meetings as needed throughout

MGB email or email used for work purposes (MGB email preferred):

MGB user ID ________

Site and Specialty Information

1) MGB clinical site(s): (select all that apply)
    a) <Sites>
2) Your medical group (select one):
    a) <Medical groups>
3) Principal care setting (where you spend the most of your time; if tied between outpatient and another setting, select outpatient). Select only one (required):
    a) Outpatient
    b) ED
    c) Inpatient
    d) Urgent Care
    e) Home Hospital
    f) Critical Care
    g) OR
    h) PACU
    i) Other
4) If applicable – other care settings. Select all that apply. optional:
    a) Outpatient
    b) ED
    c) Inpatient
    d) Urgent Care
    e) Home Hospital

© 2025 You JG et al. *JAMA Network Open*.

f) Critical Care
g) OR
h) PACU
i) Other

5) Divisions/Specialty: (dropdown)
a) Primary Care, Internal Medicine
b) Primary Care, Family Medicine
c) Primary Care, Pediatrics
d) Primary Care, Medicine / Pediatrics
e) Urgent Care
f) Hospital Medicine, Adult
g) Home Hospital
h) Hospital Medicine, Medicine / Pediatrics
i) Hospital Medicine, Pediatrics
j) Emergency Medicine, Adult
k) Emergency Medicine, Pediatrics
l) Addiction Medicine
m) Adolescent Medicine
n) Allergy and Immunology
o) Anesthesiology
p) Audiology
q) Behavioral Health
r) Breast Surgery
s) Cardiology (Interventional and Noninterventional)
t) Cardiac Surgery
u) Colorectal Surgery
v) Dermatology (including Derm Surgery)
w) Employee Health/Occupational Medicine
x) Endocrine Surgery
y) Endocrinology, Diabetes, and Metabolism
z) Gastroenterology (including Hepatology)
aa) General and GI Surgery
bb) Genetics & Genomics
cc) Geriatrics
dd) Hematology and Oncology
ee) Infectious Diseases
ff) Minimally Invasive Surgery
gg) Neonatology and Newborn Services
hh) Nephrology
ii) Neurology
jj) Neurosurgery
kk) Nutrition & Dietetics
ll) Obstetrics & Gynecology
mm) Ophthalmology

© 2025 You JG et al. *JAMA Network Open*.

nn) Oral & Maxillofacial Surgery
oo) Oral Medicine and Dentistry
pp) Orthopedic Surgery
qq) Otolaryngology (ENT, including Otology, Laryngology)
rr) Pain Medicine
ss) Palliative & Hospice Care
tt) Pediatric Allergy/Immunology
uu) Pediatric Anesthesia
vv) Pediatric Cardiology
ww) Pediatric Dermatology
xx) Pediatric Endocrinology
yy) Pediatric Gastroenterology
zz) Pediatric Genetics
aaa) Pediatric Hematology/Oncology
bbb) Pediatric Infectious Diseases
ccc) Pediatric Neurology
ddd) Pediatric Nephrology
eee) Pediatric Pulmonology
fff) Pediatric Rheumatology
ggg) Pediatric Surgery
hhh) Physical Medicine & Rehabilitation
iii) Plastic and Reconstructive Surgery
jjj) Podiatry
kkk) Psychiatry, Adult/Geriatric
lll) Psychiatry, Child/Adolescent
mmm) Pulmonology
nnn) Radiation Oncology
ooo) Rheumatology
ppp) Sleep Medicine
qqq) Sports Medicine
rrr) Surgical Oncology (Cancer Surgery)
sss) Thoracic Surgery
ttt) Trauma, Burn, Surgical and Critical Care
uuu) Transplant Surgery
vvv) Urology
www) Vascular Surgery

6) If specialty not listed (free text):
7) Primary DEP (Epic log in department), optional: free text

User Demographics

8) Clinician Title
   a) Physician (MD/DO/MBBS/MBBCh etc.)
   b) Physician Assistant
   c) Nurse Practitioner

© 2025 You JG et al. *JAMA Network Open*.

d) Other:

9) Which of the following best describes you personally?
    a) Man
    b) Non-binary
    c) Woman
    d) something else: _________
    e) I don't understand the question
    f) Prefer not to answer
10) Average Number of clinical sessions per week (if outpatient). Leave blank if not applicable:
    a) Number
11) Average Number of shifts per month (if inpatient/ED/home hospital). Leave blank if not applicable:
    a) Number
12) If you have other comments about your clinical sessions and/or shifts please indicate so here:
    a) Free text
13) How long have you been practicing medicine (since graduation from medical school, PA school, NP training, etc.)?
    a) 1-3 years
    b) 4-6 years
    c) 7-10 years
    d) 11-15 years
    e) 16-20 years
    f) >20 years
14) Smartphone operating system
    a) iPhone / Apple / iOS
    b) Android
    c) I do not have a smartphone or know I have a smartphone that is 5+ years old
    d) My clinical site has an iPad which I can use for this pilot.
    e) I have a <Vendor Z> tablet.
15) Phone Operating System Version Number (You can find this by looking at Settings > System under Android, and Settings > About under Apple/iOS). For example, if you are on iOS 17, write "iOS 17." If unsure write "Unsure"
    a) Free text
16) For Android users, device brand (e.g., Google, Samsung, etc.)
17) Cell Phone Number (to be used by our vendor to create your account). Leave blank if you are a Vendor Z user: XXX-XXX-XXXX: (free text)

Documentation Information

18) Have you used any of the following scribing services to assist in note generation (Horizontal scale of Never Used / Have previously used / Currently use)
    a) Voice to text
    b) In person scribes
    c) Remote scribes/telescribes
    d) "Virtual" human scribes
    e) AI-based Scribes

© 2025 You JG et al. *JAMA Network Open*.

19) What is the primary way (>50%) you currently document during clinic encounters, shifts, or cases?
    a) Typing directly in EHR
    b) Speech recognition
    c) Transcription
    d) In-person scribe
    e) Remote scribes/telescribes
    f) “Virtual” human scribes
    g) Other
20) Specify “Other”: ____________

B. MGB Presurvey Form Text

Hello [first_name],

Please complete the survey below by answering the questions to the best of your ability and clicking "Submit" at the bottom of the page.

If you have any questions, feel free to email us at <Email address>

1. For this pilot, I am using:
    a. Vendor X
    b. Vendor X
    c. Vendor Y
    d. Vendor Y
    e. Vendor Z
    f. Other:
2. Specify “Other”: _____________________
3. In the past month, how often did you spend time after your clinic or shift (or on days following) completing notes?
    a. Always
    b. Most sessions
    c. Some sessions
    d. Rarely
    e. Never
4. On average, about how much time do you spend per week creating and editing notes?
    a. <30 minutes
    b. 30-60 minutes
    c. 1-<2 hrs
    d. 2- <4 hrs
    e. 4- <6 hrs
    f. 6- <8 hrs
    g. > 8 hrs
5. In the past month, on average, how many minutes per week do you spend on the following tasks? (0 minutes, Less than 30 minutes, 30-60 minutes, 60-90 minutes, More than 90 minutes)

© 2025 You JG et al. *JAMA Network Open*.

    a. Completing notes during normal work hours
    b. Completing notes outside of normal work hours
6. How satisfied are you with your current method of creating and editing notes?
    a. Very satisfied
    b. Somewhat satisfied
    c. Neither satisfied nor dissatisfied
    d. Somewhat dissatisfied
    e. Very dissatisfied
7. During an average clinical day in the past 30 days, which option below most accurately represents your time spent during a clinic session/shift/work day:
    a. 80% clinical care, 20% computer-time
    b. 60% clinical care, 40% computer-time
    c. 50% clinical care, 50% computer-time
    d. 40% clinical care, 60% computer-time
    e. 20% clinical care, 80% computer-time
8. PFI Question Block
9. PFI Question Block
10. PFI Question Block
11. PWAC/Internal EHR Experience Question Block
12. PFI Question Block
13. What is your primary method for documentation in the Assessment and Plan?
    a. Problem-based charting with Epic problem list
    b. Problem-based charting free text
    c. System-based charting free text
    d. Other:
14. Specify "Other": ______________________
15. I am a qualified bilingual speaker in (choose all that apply):
    a. Arabic
    b. Chinese (Mandarin)
    c. Chinese (Cantonese)
    d. French
    e. German
    f. Korean
    g. Russian
    h. Spanish
    i. Vietnamese
    j. Haitian Creole
    k. Filipino (Tagalog)
    l. Japanese
    m. Italian
    n. Other language(s):
    o. I am not a qualified bilingual speaker.
16. Specify "Other language(s)":

© 2025 You JG et al. *JAMA Network Open*.

C. MGB Midsurvey Form Text

Hello [first_name],

Please complete the survey below by answering the questions to the best of your ability and clicking "Submit" at the bottom of the page.

If you have any questions, feel free to email us at <Email address>

1. PFI Question Block
2. PFI Question Block
3. PFI Question Block
4. PWAC/Internal EHR Experience Question Block
5. PFI Question Block
6. What would you estimate your use of AI-generated draft notes during visits has been?
    a. I have not used AI-generated draft notes
    b. Less than 10% of visits
    c. 10-24% of visits
    d. 25-49% of visits
    e. 50-74% of visits
    f. 75% or greater of visits
7. What reasons are you not using AI-generated draft notes more often? (select all that apply)
    a. Accidentally stopped recording during visits
    b. Output note not helpful for my specialty
    c. Inaccurate information in the note / significant errors
    d. Inaccurate with multi-person encounters
    e. Personal comfort with interface/technology
    f. Lack of problem based charting integration
    g. Bilingual clinician – inaccurate output without translating speech into English
    h. Interpreter use during visits – inaccurate output using interpreter during visit
    i. My patients decline recording
    j. My trust of the technology
    k. Inconvenient workflow (copy/paste into Epic)
    l. Issues with Epic integration
    m. Other
8. Specify “Other”: __________
9. How often are you seeing **significant** errors in the notes generated by AI-generated draft notes, if at all?
    a. Multiple errors in each note
    b. One error in each note
    c. One error in every four notes
    d. One error in every ten notes
    e. No major errors
    f. I have not used AI-generated draft notes enough to see these errors

© 2025 You JG et al. *JAMA Network Open*.

    g. Other:
10. Specify “Other”: _____________
11. How often are you seeing **significant errors of omission** in the notes generated by AI-generated draft notes, if at all (i.e., AI-generated draft note misses an important detail entirely)?
    a. Multiple errors in each note
    b. One error in each note
    c. One error in every four notes
    d. One error in every ten notes
    e. No errors
    f. I have not used AI-generated draft notes enough to see these errors
    g. Other:
12. Specify “Other”: _____________
13. How often are you seeing **significant errors of fact** in the notes generated by AI-generated draft notes, if at all (i.e., AI-generated draft note incorrectly adds an incorrect detail or misspells a medication name, condition or anatomic feature entirely)?
    a. Multiple errors in each note
    b. One error in each note
    c. One error in every four notes
    d. One error in every ten notes
    e. No errors
    f. I have not used AI-generated draft notes enough to see these errors
    g. Other:
14. Specify “Other”: _____________
15. For the following stylistic concerns, how often are you seeing these concerns in the AI-generated draft notes? (Multiple areas of concerns in each note/ One area of concern in each note / One area of concern in every four notes / One area of concern in every ten notes / No areas of concern / I have not used AI generated draft notes enough to see stylistic concerns)
    a. Organization of individual problems
    b. Content not in correct section of note (e.g. history in assessment and plan section instead)
    c. Irrelevant problems included
    d. Phrasing of clinical content
    e. Phrasing of non-clinical content
    f. Language that is not patient-centric
    g. Organization of particular sections (e.g. history of present illness)
16. Please share any other comments about errors or stylistic concerns not mentioned above:
    a. <free text>
17. What is your trust in AI-generated draft notes?
    a. I strongly trust the technology
    b. I somewhat trust the technology
    c. I somewhat do not trust the technology
    d. I strongly do not trust the technology
18. If 'strongly do not trust' or 'somewhat do not trust', please explain why not: (free text)
19. Other feedback (positive or negative): free text

D. MGB Postsurvey Form Text

Hello [first_name],

Thank you for participating in the pilot! As a last step, please complete the survey below by answering the questions

to the best of your ability and clicking "Submit" at the bottom of the page.

If you have any questions, feel free to email us at <Email address>

For the questions that follow – AI generated draft notes include notes generated with any of the following products: Vendor X, Vendor Y, or Vendor Z.

1. Do you feel using AI generated draft notes has impacted the quality of your clinical note?
    a. Yes, significant improvement
    b. Yes, some improvement
    c. Yes, somewhat worse
    d. Yes, significantly worse
    e. No change
2. Do you feel that using AI generated draft notes was able to save you time?
    a. Yes
    b. No
    c. Maybe
3. I would estimate that using AI generated draft notes during a clinical session affected my efficiency in the following way:
    a. Much more efficient
    b. Somewhat more efficient
    c. Minimally more efficient
    d. No more efficient
    e. Less efficient
4. Were you satisfied with the AI generated draft notes turnaround time?
    a. Very satisfied
    b. Somewhat satisfied
    c. Neither satisfied nor dissatisfied
    d. Somewhat dissatisfied
    e. Very dissatisfied
5. Have you seen any change in patient experience while utilizing AI generated draft notes? (check any that apply)
    a. Increased provider/patient face time
    b. Decreased provider/patient face time
    c. Increased quality of provider/patient interaction
    d. Decreased provider/patient interaction
    e. Increased patient wait times
    f. Reduced patient wait times
    g. Increased patients leaving without being seen

© 2025 You JG et al. *JAMA Network Open*.

h. Reduced patients leaving without being seen
i. Increased perceived patient satisfaction with visit
j. Decreased perceived patient satisfaction with visit
k. None of the above
l. Other

6. Specify “Other”: ________
7. How would you rank the following features: (Very Satisfied / Somewhat satisfied / Neither satisfied nor dissatisfied / Somewhat dissatisfied / Very dissatisfied)
   a. Recording sessions
   b. Task of uploading recording
   c. Time for note to be generated
   d. Notification when note is uploaded
   e. Quality of the initial note
   f. Ability to make edits to the note
   g. Quality of the final note
8. On a scale of 1-10, how likely are you to recommend this application to another clinician? (10 being highly likely) (1-10 number scale, 1 Highly Unlikely 10 Highly Likely) - Place a mark on the scale.
9. Are there features of the technology you felt strongly about that you would like to comment on?
10. Indicate if you had any technical issues using AI generated draft notes. If so, indicate whether you were satisfied with your interaction with customer support.
11. Please answer how much you agree/disagree with the following statements. (Strongly Agree / Somewhat Agree / Neither Agree Nor Disagree / Somewhat Disagree / Strongly Disagree / Not Applicable)
    a. As a provider, knowing that a practice/clinic/ site utilizes AI for clinical documentation would positively influence my decision to choose to work at that facility/institution.
    b. Knowing that a practice / clinic / site utilizes AI for clinical documentation would positively influence my decision to remain practicing at my current location.
    c. When using AI tools for clinical documentation, I am able to give more individual attention to my patient during their visit/encounter/interaction.
    d. Using AI clinical documentation applications allowed more time for teaching.
    e. If I could continue to use AI generated draft notes in the future, I would be more likely to extend the length of my clinical career in medicine.
12. How would you feel if AI generated draft notes applications were no longer available?
    a. Very disappointed
    b. Disappointed
    c. Indifferent
    d. Satisfied
    e. Very Satisfied
    f. Other
13. Specify “Other”: ________
14. In the past month, on average, how many minutes per week did you spend on the following tasks? (0 minutes, Less than 30 minutes, 30-60 minutes, 60-90 minutes, More than 90 minutes)
    a. Completing notes during normal work hours
    b. Completing notes outside of normal work hours
15. PFI Question Block

16. PFI Question Block
17. PFI Question Block
18. PWAC/Internal EHR Experience Question Block
19. PFI Question Block
20. How often do you estimate you have used the AI generated draft notes?
    a. Only once
    b. Less than once per clinic session
    c. Once per clinic session
    d. 25% of visits per session
    e. 50% of visits per session
    f. Every patient encounter per clinic session
21. What reasons are you not using AI generated draft notes more often? (select all that apply)
    a. Accidentally stopped recording during visits
    b. Output note not helpful for my specialty
    c. Inaccurate information in the note / significant errors
    d. Inaccurate with multi-person encounters
    e. Personal comfort with interface/technology
    f. Lack of problem based charting integration
    g. Bilingual clinician – inaccurate output without translating speech into English
    h. Interpreter use during visits – inaccurate output using interpreter during visit
    i. My patients decline recording
    j. My trust of the technology
    k. Inconvenient workflow (copy/paste into Epic)
    l. Other: free text comment
22. How often did you see **significant errors** in the notes generated by AI generated draft notes, if at all?
    a. Multiple errors in each note
    b. One error in each note
    c. One error in every four notes
    d. One error in every ten notes
    e. No major errors
    f. I have not used AI generated draft notes enough to see these errors
    g. Other
23. Specify "Other": ________
24. How often did you see **significant errors of omission** in the notes generated by AI generated draft notes, if at all (i.e., the software misses an important detail entirely)?
    a. Multiple errors in each note
    b. One error in each note
    c. One error in every four notes
    d. One error in every ten notes
    e. No errors
    f. I have not used AI generated draft notes enough to see these errors
    g. Other: free text comment

25. How often did you see **significant errors of fact** in the notes generated by AI generated draft notes, if at all (i.e., the software incorrectly adds an incorrect detail or misspells a medication name, condition or anatomic feature entirely)?
    a. Multiple errors in each note
    b. One error in each note
    c. One error in every four notes
    d. One error in every ten notes
    e. No errors
    f. I have not used AI generated draft notes enough to see these errors
    g. Other
26. Specify “Other”: ________
27. For the following stylistic concerns, how did you see these concerns in the AI-generated draft notes? (Multiple areas of concerns in each note/ One area of concern in each note, one area of concern in every four notes / one area of concern in every ten notes / no areas of concern / I have not used AI generated draft notes enough to see stylistic concerns)
    a. Organization of individual problems
    b. Content not in correct section of note (e.g. history in assessment and plan section instead)
    c. Irrelevant problems included
    d. Phrasing of clinical content
    e. Phrasing of non-clinical content
    f. Language that is not patient-centric
    g. Organization of particular sections (e.g. history of present illness)
28. Please share any other comments about errors or stylistic concerns not mentioned above:
    a. <free text>
29. What is your trust level in AI generated draft notes technology?
    a. I strongly trust AI generated draft notes technology
    b. I somewhat trust AI generated draft notes technology
    c. I somewhat do not trust AI generated draft notes technology
    d. I strongly do not trust AI generated draft notes technology
30. Do you have any other feedback related to using AI generated draft note applications or the pilot? (please provide below)

E. Emory Presurvey Form Text

<Vendor> Onboarding Registration

Thank you so much for participating in our <Vendor> program - our goal is to alleviate the burden of documentation to help you concentrate on what's more important: your patients.

To help gauge if we are meeting our goal, we would like you to register and take a brief survey to understand your current perception of how you document your visits.

This survey should take less than 10 minutes to complete. We appreciate your time and input as we aim to deliver you a better Digital experience!

1. Please enter your Emory email

© 2025 You JG et al. *JAMA Network Open*.

Please rank your agreement to the following statements

2. Prior to <Vendor>, my documentation process met my requirements as a provider.
- Strongly Agree
- Agree
- Neutral
- Disagree
- Strongly disagree

3. Prior to <Vendor>, my documentation process was easy for me to complete
- Very negative
- Negative
- Neutral
- Positive
- Very positive

4. Prior to <Vendor>, my documentation process had the following impact on my wellbeing
- Very negative
- Negative
- Neutral
- Positive
- Very positive

5. Prior to <Vendor>, my documentation process had the following impact on the patient experience
- Very negative
- Negative
- Neutral
- Positive
- Very positive

F. Emory Postsurvey Form Text

<Vendor> Follow-Up Survey

Thank you so much for participating in our <Vendor> program - our goal is to alleviate the burden of documentation to help you concentrate on what's more important: your patients.

To help gauge if we are meeting our goal, we would like you to take a brief follow-up survey to understand your perception of how <Vendor> is working for you.

© 2025 You JG et al. *JAMA Network Open*.

This survey should take less than 10 minutes to complete. We appreciate your time and input as we aim to deliver you a better Digital experience!

1. Please enter your Emory email

Please rank your agreement to the following statements

2. How likely are you to recommend <Vendor> to a colleague or coworker?

[0, 1, 2, 3, 4, 5, 6, 7, 8, 9, 10] (Not at all likely – 0; Extremely likely –10)

3. Using <Vendor> increases my productivity

- Strongly agree
- Agree
- Neutral
- Disagree
- Strongly disagree

4. My current documentation process meets my requirements as a provider.

- Strongly agree
- Agree
- Neutral
- Disagree
- Strongly disagree

5. My current documentation process is easy for me to complete

- Strongly agree
- Agree
- Neutral
- Disagree
- Strongly disagree

6. My current documentation process has the following impact on my wellbeing

- Very negative
- Negative
- Neutral
- Positive
- Very positive

7. My current documentation process has the following impact on the patient experience

© 2025 You JG et al. *JAMA Network Open*.

- Very negative
- Negative
- Neutral
- Positive
- Very positive

8. I use <Vendor> for:

- All of my notes
- Most of my notes
- Some of my notes
- Few of my notes
- None of my notes

9. If Some/Few/None, why?

<Freetext box>

© 2025 You JG et al. *JAMA Network Open*.
