# Supplementary material for: Ambient Documentation Technology in Clinician Experience of Documentation Burden and Burnout
Source: JAMA Netw Open. 2025 Aug 21;8(8):e2528056. doi: 10.1001/jamanetworkopen.2025.28056 (PMC12371510; doi:10.1001/jamanetworkopen.2025.28056)
Supplement: Supplement 2. — Data Sharing Statement [file jamanetwopen-e2528056-s002.pdf]

Data Sharing Statement

You. Ambient Documentation Technology in Clinician Experience of Documentation Burden and Burnout. *JAMA Netw Open*. Published August 21, 2025.
doi:10.1001/jamanetworkopen.2025.28056

Data
Data available: No

Additional Information
Explanation for why data not available: This is proprietary health system data.
